# Active natural compounds perturb the melanoma risk-gene network

**DOI:** 10.1093/g3journal/jkad274

**Published:** 2023-11-30

**Authors:** Luying Shao, Yibo Zhao, Michael Heinrich, Jose M Prieto-Garcia, Claudia Manzoni

**Affiliations:** Department of Pharmaceutical and Biological Chemistry, UCL School of Pharmacy, WC1N 1AX London, UK; Department of Pharmacology, UCL School of Pharmacy, WC1N 1AX London, UK; Department of Pharmaceutical and Biological Chemistry, UCL School of Pharmacy, WC1N 1AX London, UK; Chinese Medicine Research Center, and Department of Pharmaceutical Sciences and Chinese Medicine Resources, College of Chinese Medicine, China Medical University, Taichung City 404333, Taiwan; School of Pharmacy and Biomolecular Sciences, Liverpool John Moores University, L3 3AF Liverpool, UK; Department of Pharmacology, UCL School of Pharmacy, WC1N 1AX London, UK

**Keywords:** melanoma, network analysis, harmine, berberine chloride, differential gene expression

## Abstract

Cutaneous melanoma is an aggressive type of skin cancer with a complex genetic landscape caused by the malignant transformation of melanocytes. This study aimed at providing an in silico network model based on the systematic profiling of the melanoma-associated genes considering germline mutations, somatic mutations, and genome-wide association study signals accounting for a total of 232 unique melanoma risk genes. A protein–protein interaction network was constructed using the melanoma risk genes as seeds and evaluated to describe the functional landscape in which the melanoma genes operate within the cellular milieu. Not only were the majority of the melanoma risk genes able to interact with each other at the protein level within the core of the network, but this showed significant enrichment for genes whose expression is altered in human melanoma specimens. Functional annotation showed the melanoma risk network to be significantly associated with processes related to DNA metabolism and telomeres, DNA damage and repair, cellular ageing, and response to radiation. We further explored whether the melanoma risk network could be used as an in silico tool to predict the efficacy of anti-melanoma phytochemicals, that are considered active molecules with potentially less systemic toxicity than classical cytotoxic drugs. A significant portion of the melanoma risk network showed differential expression when SK-MEL-28 human melanoma cells were exposed to the phytochemicals harmine and berberine chloride. This reinforced our hypothesis that the network modeling approach not only provides an alternative way to identify molecular pathways relevant to disease but it may also represent an alternative screening approach to prioritize potentially active compounds.

## Introduction

Cutaneous malignant melanoma (CMM, hereafter referred to as melanoma) is the most aggressive type of skin cancer caused by the malignant transformation of melanocytes. In a stepwise process known as melanomagenesis, melanocytes acquire the ability to replicate in an uncontrolled fashion before leaving their original site and migrating through the lymphatic system. Melanoma is among the most frequently diagnosed cancers in the United Kingdom and in the United States of America and, over the past decade, it showed increasing incidence rates (Memon et al. 2021; Cancer Research UK).

Melanoma is diagnosed and classified based on the morphological appearance of the skin pigmented lesion (border shape, color, dimension, thickness, ulcerations, etc.), presence/absence of metastases and molecular assessment of the tumor and sentinel lymph node biopsies (Amin et al. 2017). The current therapeutic approach comprises the surgical excision of the tumor in combination with local/systemic anticancer treatment to target metastases (radiation therapy, chemotherapeutics, and more specific molecular treatments targeting genes involved in cell proliferation) (Pathak and Zito 2022).

As for all cancer types, melanoma is a complex disease with a multifactorial etiopathogenesis where the cause is a complex combination of environmental exposures and genetic-risk factors. The most relevant environmental risk factor for melanoma is exposure to UV radiation as this causes both DNA damage with accumulation of mutations and oxidative stress with consequent inflammation of the skin tissues (Sample and He 2018).

An average of ∼10% of melanoma cases show a positive familial history (of pure melanoma or mixed cancer syndromes) thus suggesting the existence of large effect size heritable mutations capable of causing melanoma (Soura et al. 2016). The locus most frequently mutated in familial melanoma (accounting for ∼20% of familial cases) is *CDKN2A* (Goldstein and Tucker 2001, Goldstein et al. 2006) which encodes for 2 different proteins (CDN2A/p16^INK4^/MTS1/CDK4I and p14ARF) (Haluska et al. 2006). However, other genes are affected by rare to very rare mutations and some of the melanoma heritability is still not fully understood. A second type of germline genetic alterations that contribute to melanoma is small effect size variants. These variants act more as risk factors rather than causal mutations. Due to their small effect size, they cannot be linked to melanoma with the classical studies of families; in fact, as they do not cause disease *per se*, they do not appear to segregate with disease. These small effect size risk variants are investigated via genome-wide association studies (GWAS) (Landi et al. 2020). Unfortunately, only a small percentage of these risk variants are coding, the vast majority is noncoding (intronic or intergenic) meaning that it is difficult to understand, with certainty, which gene(s) is eventually responsible for their molecular effect on increasing the risk of melanoma (Manzoni et al. 2020).

Germline mutations, both in the form of large effect size mutations and small effect size risk variants, are therefore associated, to different extents, with an increased individual risk of melanoma. Alongside these germline mutations, somatic mutations play a crucial role in both the triggering and the development of melanoma. Somatic mutations (both UV-dependent and UV-independent) can start melanomagenesis, while their accumulation in the transformed cells, as consequence of uncontrolled cell replication, can influence cancer progression. Some of the genes that are most frequently affected by somatic mutations are *BRAF*, *NRAS*, and *TP53* (Sample and He 2018) however a plethora of other genes can harbor somatic mutations in melanoma.

The complex genetic landscape of melanoma has prompted us to investigate it via in silico modeling. Systems biology can, in fact, provide a systematic approach to evaluate all the genes involved in a certain complex disease together as a functional unit rather that as single entities considered in isolation. The approach has therefore the advantage of generating an in silico model of the global, genetic-risk landscape of melanoma. This model can be used to evaluate, as a group, all the genes contributing to the risk of melanoma, defining functional units of tightly connected genes that are likely to work together to sustain communal pathways relevant to melanoma onset and progression. In this work, we assessed how perturbations of these relationships across the melanoma genes might be used to screen the effect of (potentially therapeutic) phytochemicals in a melanoma setting. To exemplify how the in silico risk-model of melanoma can be used for such studies, we selected 2 natural compounds, harmine and berberine chloride, that are reported in literature for their anticancer properties and are suggested to have an effect on melanoma albeit their precise mechanisms of action not being completely clear. Harmine is a β-carboline alkaloid known for its antiviral activity (Hudson et al. 1986) and anti-inflammatory effect achieved via inhibition of NF-kB signaling (Liu et al. 2017). The antitumor effect of harmine was evaluated in different models; its antimetastatic effect was reported in vitro and in vivo in melanoma models and showed to be mediated by down-regulation of pro-metastatic genes (such as MMP-9, ERK, and VEGFs) (Hamsa and Kuttan 2011a). Harmine is also supposed to modulate apoptosis and regulate various transcription factors (Hamsa and Kuttan 2011b). Furthermore, harmine was able to significantly reduce the levels of pro-angiogenic factors, capillary formation, and microvessel outgrowth in different models (Hamsa and Kuttan 2010) thus suggesting harmine to be an antiangiogenic compound. Berberine is a benzylisoquinoline alkaloid mainly extracted from members of the genus *Berberis*. This phytochemical is known for many activities (Neag et al. 2018) ranging from nephroprotection (Liu et al. 2008) and glucose control during diabetes (Chen et al. 2011) to modulation of cholesterol levels (Kong et al. 2008). Berberine activity has been studied in different cancer models showing anticarcinogenic properties achieved via regulation of cell cycle, activation of caspases and autophagy, alteration of gene expression, and many other mechanisms (Wang et al. 2020). Recently, berberine was shown to be active in reducing number and mobility of human melanoma cells (Liu et al. 2018). We verified that these active compounds were indeed able to significantly alter the expression of genes topologically relevant for the in silico risk-model of melanoma.

## Materials and methods

### Gene profiling

Melanoma-associated genes were collected in 3 ways including: (1) genes with familial (germline) mutations typically associated with melanoma (referred to as “familial genes”), (2) genes known to develop somatic mutations within the growing melanoma cells (referred to as “somatic-mutations genes”), and (3) genes prioritized based on GWAS single nucleotide polymorphisms (SNPs) associated with increased risk of melanoma (referred to as “risk genes”).

Genes whose mutations are associated with familial melanoma are widely studied therefore the list of familial genes was derived from a comprehensive and updated review paper published in 2020 (Toussi et al. 2020).

The list of genes shown to accumulate somatic mutations within the melanoma tissue was sourced from the catalogue of somatic mutations in cancer (COSMIC v95, https://cancer.sanger.ac.uk/cosmic; RRID:SCR_002260) (Tate et al. 2019). COSMIC cancer browser was used to select skin data, including all sub-tissue types. The histology selection was focused on “Benign Melanocyte Nevus” (all sub-histology included) and “Malignant Melanoma” (all sub-histology included). Finally, the “All screens” filter type was selected to collect the top-20 mutated genes (considering total number of samples harboring a mutation in the gene) in each of the 2 conditions (data downloaded in March 2022). The Cancer Genome Atlas by the National Cancer Institute (TCGA, https://www.cancer.gov/about-nci/organization/ccg/research/structural-genomics/tcga, March 2022, RRID:SCR_003193) was used to complement the COSMIC database search. Publications from the TCGA were screened in reference to skin cutaneous melanoma; only 1 marker paper was available in the “TCGA Cancers Selected for Study” resource (Akbani et al. 2015). Within this publication, genes carrying somatic mutations (42 genes) as well as genes identified as frequently involved in fusion events (*AKT3*, *BRAF*, *HMGA2*, *MITF*, and *RAF1*) were selected.

**Fig. 1.**
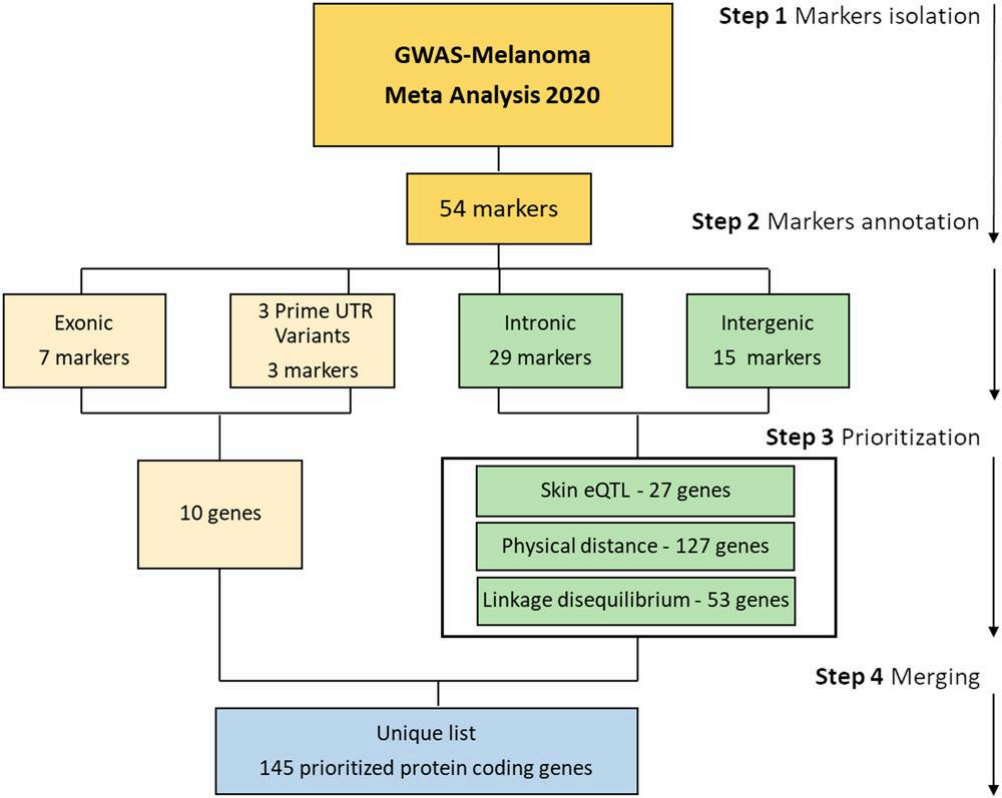
Gene prioritization pipeline. Workflow for the isolation of candidate genes associated with the risk of sporadic melanoma (GWAS genes). All the steps are detailed in the main text. eQTL analysis was conducted for each SNP using the Genotype-Tissue Expression (GTEx, RRID:SCR_001618) Dataset (GTEx_Analysis_v8, https://gtexportal.org/) and collecting data for skin sun-exposed and skin non-sun-exposed eQTLs.

The list of GWAS risk SNPs was obtained from (Landi et al. 2020). Four different approaches were used to prioritize protein coding genes around the risk signals thus obtaining the list of melanoma risk genes. A summary of the workflow for risk gene prioritization is shown in Fig. 1. Briefly, SNPs associated with risk of cutaneous melanoma were collected from the 2020 meta-analysis (Landi et al. 2020). The genomics position of each SNP was annotated using the genomic built GRCh37 using dbSNP (https://www.ncbi.nlm.nih.gov/snp/) (Sherry et al. 2001). Each SNP was further cataloged in (1) coding if the SNP was in an exon of a coding open reading frame (ORF); (2) intronic if the SNP was located in an intron of a coding ORF; (3) intergenic—if the SNP was not located in a coding ORF. All the coding SNPs were listed as associated with the gene in which they were contained. In contrast, for each of the noncoding (intronic and intergenic) SNPs, the list of potentially associated genes was compiled after analysis of the locus drawn around the SNP as well as analysis of its expression quantitative trait loci (eQTL) in skin. In more details, the following 2 strategies (physical distance = PD and linkage disequilibrium = LD) were used to design a locus around each (intronic and intergenic) marker. In the PD method, the locus was defined as a window of ±250 kb (upstream and downstream) around the risk marker. In the LD method, the locus was defined by the window containing all SNPs in *r*^2^ ≥ 0.8 with the risk marker. In both cases (PD and LD), the loci were drawn using SNPsnap (https://bioinformaticshome.com/tools/descriptions/SNPsnap.html#gsc.tab=0) (Pers et al. 2015), using 1000 Genome, European population as a reference and excluding automatic annotations for the HLA locus. The genomic elements within the loci drawn as detailed above were extracted and listed using their Ensemble Gene (ENSG) identifier. These identifiers were converted to the HUGO Gene Nomenclature Committee (HGNC) approved gene symbols (Bateman et al. 2021) using g:Profiler (g: Converter, https://biit.cs.ut.ee/gprofiler/convert) (Raudvere et al. 2019). After gene symbol conversion, the genomic elements containing the following keywords within their names “nan”, “none”, “pseudogene”, “novel”, “antisense”, “microRNA”, “IncRNA”, “small nucleolar RNA”, “yRNA”, “TEC”, “readthrough”, “intergenic”, and “intronic” were excluded thus obtaining a final list of protein coding ORFs/genes.

### Protein–protein interactions study

The Human Integrated Protein-Protein Interaction rEference (HIPPEv2.3) (http://cbdm-01.zdv.uni-mainz.de/∼mschaefer/hippie/network.php) (Alanis-Lobato et al. 2017) network query tool was used to download protein–protein interactions (PPIs) using the melanoma genes as seeds. HIPPIE (RRID:SCR_014651) is a regularly updated resource that allows for the download of protein interactions from several primary databases in which PPIs are collected from peer-reviewed literature. Interactions were downloaded at “level zero” meaning across the melanoma genes only (without any intermediate bridge) and at “level 1” meaning all the direct PPIs of the melanoma genes. Data were downloaded in June 2022. For the “level zero”, all interactions were downloaded and quality control (QC) was carried out manually over the entire set of downloaded interactions considering: (1) the PubMed ID (interactions that did not present with a publication identifier were discarded); and (2) the interaction detection method (interactions with no interaction detection method or with an “unspecified” interaction detection method only (Supplementary Table 1) were considered “low quality” and discarded). For the “level 1” analysis, QC was skipped as only data with a confidence value above 0.72 (high confidence) were collected thus allowing for the download of interactions with the largest confidence score (most likely to be reproducible based on the methods used for their identification, the number of publications reporting the interaction and the reproducibility in organisms other than human (Schaefer et al. 2012)); however, UBB, UBC, and UBD interactions were removed as considered unspecific; melanoma genes interactors mapped to multiple Entrez gene IDs were removed as well as melanoma genes interactors with no standard HGNC gene symbol. Of note, since the gene symbol has been used as identifier, CDKN2A interactions for CDN2A(P42771) and ARF(Q8N726) are combined. For cluster analysis, the level 1 network was processed in Cytoscape using the Fast-Greedy clustering algorithm app.

### Gene ontology enrichment analysis

Functional enrichment was performed using the g:Profiler server (Raudvere et al. 2019) (g:GOSt tool, https://biit.cs.ut.ee/gprofiler/gost, RRID:SCR_006809) in May/June; December 2022 or September 2023. Gene ontology biological process (GO-BPs; RRID:SCR_002811) terms were selected (no electronic GO annotations). The significant GO-BPs retrieved were further processed and terms with large “term size” (the specific threshold is specified in each experiment) were removed, effectively keeping only the most specific GO-BP terms in the enrichment analysis. GO-BPs were further grouped into semantic classes based on: (1) semantic similarity using an in-house pipeline (as shown in the additional column added to the g:Profiler results in each of the supplementary tables referring to GO-BPs enrichment), this pipeline entails the identification of keywords within the enriched GO:BP term names and further dimension reduction of the dataset of enriched terms by grouping terms using the identified keywords; or (2) hierarchical relations retrieved via the R package GO.db.

### Reactome pathways enrichment analysis

Functional enrichment was performed using the g:Profiler server (Raudvere et al. 2019) (g:GOSt tool, https://biit.cs.ut.ee/gprofiler/gost, RRID:SCR_006809) in September 2023. Reactome pathway (Gillespie et al. 2022) (RRID:SCR_003485) terms were selected. The significant pathways retrieved were further processed and terms with “intersection size” ≤ 3 were removed, considering that we would need more than 3 proteins to map to a pathway. Pathways were further grouped into semantic classes based on semantic similarity as shown in the additional column added to the g:Profiler results (presented in the Supplementary materials).

### Differential expression analysis

Harmine is a beta-carboline alkaloid, naturally isolated from several medicinal plants such as *Peganum harmala* L. (wild rue, Nitrariaceae) and *Banisteriopsis caapi* (Spruce ex Griseb.) Morton (ayahuasca, Malpighiaceae). Berberine chloride as isoquinoline alkaloid is mainly extracted from the plants members of the genus *Berberis*. Harmine and berberine chloride are widely studied phytochemicals and therefore they are both commercially available (Sigma, 286044; Sigma, B3251).

SK-MEL-28 human melanoma cells were plated (8.5 × 10^5^) in complete growing medium (MEM with GlutaMax + 10% fetal bovine serum + 1% nonessential amino acid + 1 mM sodium pyruvate) and allowed to adhere overnight (37°C, 5% CO_2_). Cells were exposed to the treatment (harmine 43 μM, berberine chloride 25 μM, or the vehicle 0.43% DMSO) for 24 hours before harvesting. Cells were washed 3 times with PBS and collected with trypsin. Cell pellets were immediately frozen and stored at −80°C. RNA extraction and RNA sequencing were performed at UCL genomics (Kapa mRNA Hyper Prep, Illumina NextSeq 2000 with 16 M reads per sample). Three independent replicates were sequenced per condition. Differential expression analysis (DEA) was performed at UCL genomics using the SARTools package with the DESeq2 pipeline (Love et al. 2014). Hierarchical clustering was performed on the normalized read counts using the ComplexHeatmap R package (Gu et al. 2016).

### Programs and IT resources

PPI networks and GO-BP hierarchical network were drawn using Cytoscape (Version 3.8.2, RRID:SCR_003032) (Shannon et al. 2003); databases were parsed using Excel spreadsheets and R-studio (RStudio 1.4 1103). Relevant scripts are available as Supplementary File 1.

## Results

### Melanoma gene profiling and selection

A total of 54 SNPs associated with risk of cutaneous melanoma were suggested in the 2020 GWAS meta-analysis (Landi et al. 2020); 19 of these markers have been replicated from previous studies (Law et al. 2015), while the other 35 loci were identified as new associations.

Annotation of the 54 markers showed that only 10 out of 54 SNPs were in exonic regions or in the 3′ untranslated region (3′ UTR) of protein coding genes. The remaining 44 SNPs were either intergenic or intronic. In both cases, these SNPs were considered noncoding (Supplementary Table 2). The 10 exonic and 3′ UTR SNPs were considered to produce a functional change in the protein coding genes in which they were contained; while a list of potentially affected protein coding ORFs was compiled for the 44 noncoding (intronic and intergenic) SNPs by considering physical distance (PD), linkage disequilibrium (LD), and skin eQTLs (Fig. 1).

One marker (rs12984831) was not present in dbSNP, nor a proxy was available; therefore, this marker was not annotated for LD but only (manually) for PD. One marker (rs28986343) was present in the HLA region; as this is a complex region to annotate for LD, this marker was only annotated with PD measures.

After annotation of all markers, we produced a final list of genes (hereafter referred to as “GWAS genes”) containing: 10 genes (corresponding to the protein coding genes containing the coding risk SNPs) and 135 prioritized genes found in: (1) the LD blocks with markers in *r*^2^ ≥ 0.8 with the noncoding SNPs (*n* = 53) and/or (2) in the loci drawn around the noncoding, risk SNPs in a physical window of ±250 kb (*n* = 127); and/or (3) in eQTL with the noncoding SNPs in sun-exposed and/or non-sun-exposed skin (*n* = 27). (Supplementary Table 3). Of note, 18.5% of the 135 prioritized genes (25/135) were found in eQTL as well as in proximity and/or LD with the risk SNP, while only 1.5% of the genes (2/135) were found only in eQTL with the risk SNP; finally, the vast majority, 80% of the genes (108/135), were only found in proximity and/or LD with the risk SNP but not in eQTL.

This list of melanoma genes derived from the prioritization of the GWAS study was supplemented with 27 additional genes whose mutations are known to be causative of familial melanoma or melanoma-subordinate syndromes (hereafter referred to as “familial genes”) as reported in Toussi et al. (2020) (Supplementary Table 3).

Finally, COSMIC was interrogated to collate the list of top 20 genes most frequently mutated in “Benign Melanocyte Nevus” and “Malignant Melanoma” specimens irrespectively of the location of the skin biopsy (all skin sub-tissue types were included). After removal of genes duplicated across the 2 conditions, a list of 34 unique genes was obtained. Given the complexity of prioritizing genes harboring somatic mutations, the list of mutated genes as per the COSMIC repository was complemented with an additional list obtained from a sequencing project lead by the TCGA and run on a set of cutaneous primary and/or metastatic melanomas (Akbani et al. 2015). This led to a total of 71 unique genes reported as frequently mutated within melanoma (hereafter referred to as “somatic genes”) (Supplementary Table 3).

**Fig. 2.**
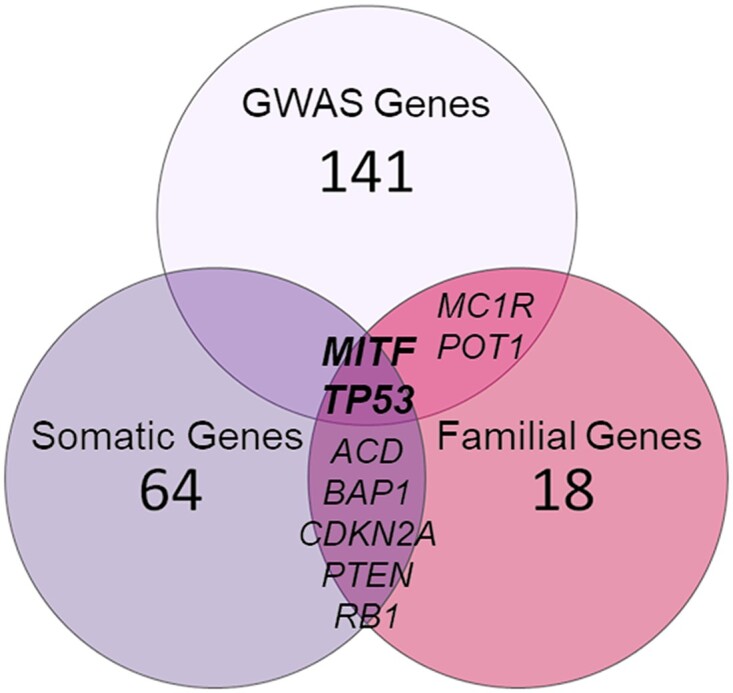
Melanoma-gene list. Venn diagram describing the composition of the final melanoma risk gene list.

The 3 lists of melanoma-associated genes: (1) GWAS genes (*n* = 145), (2) familial genes (*n* = 27), and (3) somatic genes (*n* = 71) were merged into the final “melanoma risk genes” list (Supplementary Table 3) for further analyses. This final list contained a total of 232 unique genes; only 2 genes (*MITF* and *TP53*) were communal to the 3 lists (i.e. GWAS, familial and somatic genes), while 7 genes (∼3%) were prioritized from 2 lists (Fig. 2). All other 223 genes were present in 1 list only.

### Functional validation of the melanoma risk gene list

**Fig. 3.**
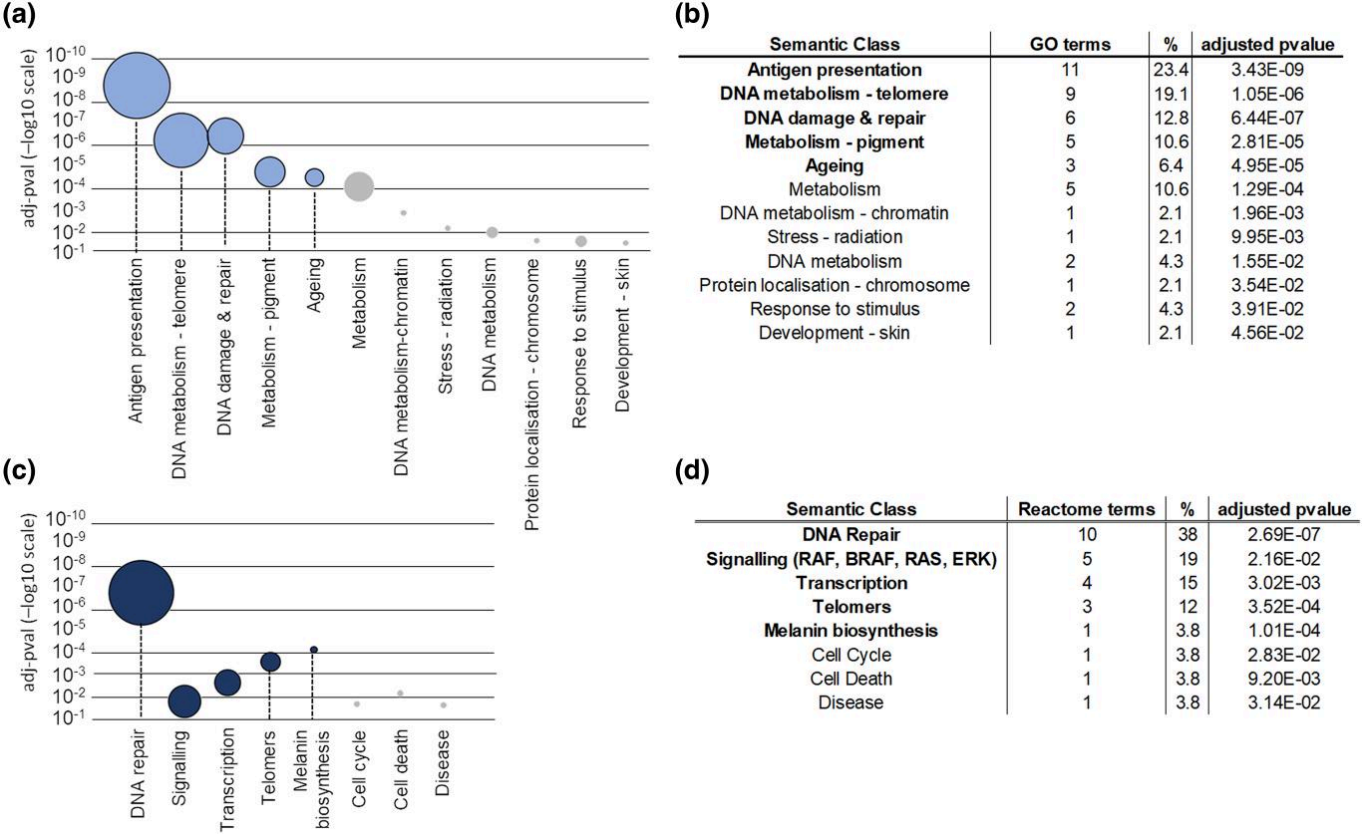
Functional enrichment of the melanoma-gene list. a) Semantic classes are listed on the *x*-axis, the position of the circle on the *y*-axis represents the adjusted *P*-value of the most significant GO-BP term enriched in the semantic class (axis in −log10 scale). The width of the circle is proportional to the number of GO-BPs that are grouped within the semantic class. b) Table reporting details of the semantic classes; GO terms, number of GO terms grouped in the semantic class; %, percentage of terms involved in the semantic class in comparison with the total number of enriched GO terms; adjusted pvalue, g:SCS *P*-value of the most significantly enriched GO term within the semantic class. GO:BP terms were filtered by term size < 100. c) Semantic classes are listed on the *x*-axis, the position of the circle on the *y*-axis represents the adjusted *P*-value of the most significant Reactome term enriched in the semantic class (axis in −log10 scale). The width of the circle is proportional to the number of Reactome pathways that are grouped within the semantic class. d) Table reporting details of the semantic classes; Reactome terms, number of pathway terms grouped in the semantic class; %, percentage of terms involved in the semantic class in comparison with the total number of enriched Reactome terms; adjusted pvalue = g:SCS *P*-value of the most significantly enriched Reactome term within the semantic class.

The 232 melanoma risk genes were annotated via functional enrichment analysis using 2 different ontologies of reference: Gene Ontology biological processes (GO:BPs) and Reactome pathways. After QC, the GO-BPs were semantically grouped into 12 semantic classes (Supplementary Table 4, Fig. 3). The most relevant semantic classes (in terms of adjusted *P*-value and/or number of GO-BPs) were: “antigen presentation”, “metabolism of telomeres”, “DNA damage & repair”, “metabolism of pigments”, and “ageing”. With the exception of the “antigen presentation” class, the enrichment clearly pointed toward biological processes expected to be relevant in cancer in general (DNA damage and repair, telomeres, and ageing) and in melanoma in particular (metabolism of pigments). Similarly, Reactome pathway terms were also grouped by semantic similarity (Supplementary Table 5, Fig. 3) into 8 groups of terms referring to: “DNA repair”, “signalling via RAF, BRAF, RAS and ERK”, “transcription”, “telomer associated pathways”, “melanin biosynthesis”, “cell cycle”, “cell death”, and the very general term “disease”. It is interesting to notice that in both cases the top enriched terms contained reference to: DNA repair, ageing/telomeres, and melanin/pigment metabolism/biosynthesis. Antigen presentation was only capture by GO:BPs (not by pathways in Reactome) while RAS/BRAF/RAF/ERK signaling and transcription were only captured by Reactome (not by GO:BPs). These discrepancies between GO:BPs and Reactome pathways enrichment are probably due to the different ontologies of reference, however there was a large overlap in the final results showing the genes prioritized in the melanoma-gene list are indeed involved in biological processes expected to be relevant in cancer and in melanoma.

### The melanoma-gene protein network

**Fig. 4.**
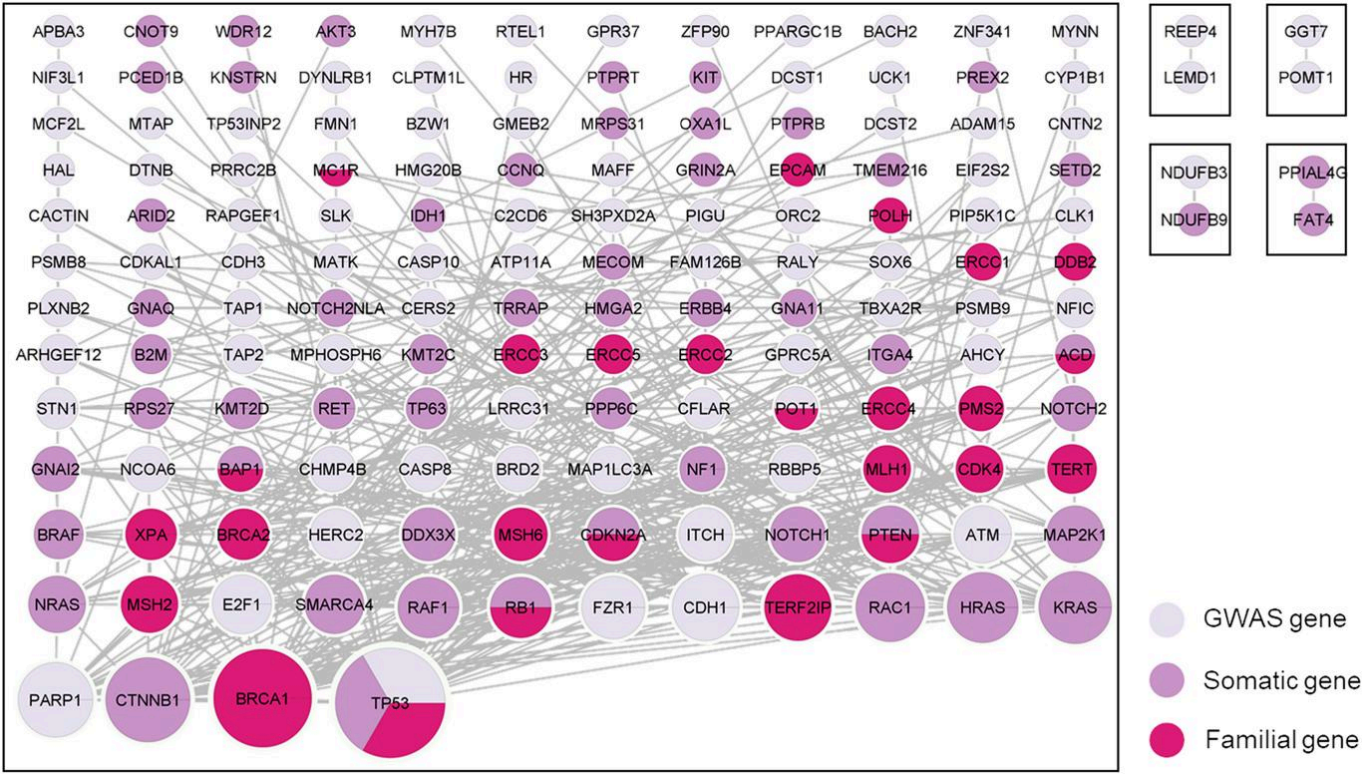
PPI network (level-zero interactions). Nodes are color-coded as per figure legend indicating protein products of GWAS (light purple), somatic (purple), and familial (pink) genes. The size of nodes is proportional to the number of connections (=number of edges) connecting the node to the network. Picture drawn with Cytoscape, grid layout.

PPIs directly connecting each of the protein products of the melanoma risk genes (level-zero PPIs) were extracted from peer-reviewed literature using the HIPPE web application. After QC, removal of self-interactions, and plotting of the pairwise protein interactions across the melanoma risk genes, the level-zero graph was composed of 1 main graph defining a dense, interconnected network containing the majority of the melanoma risk proteins and 4 independent units describing pairwise connections (NDUFB3:NDUFB9; GGT7:POMT1; REEP4:LEMD1; PPIAL4G:FAT4) (Fig. 4, Supplementary Table 6). We labeled the larger and interconnected graph as “core-level-zero PPI network” as it contains the protein products of 148 out of the 232 melanoma risk genes (63.7%) and clearly shows they are directly connected with each other. Within the core-level-zero PPI network, 19 proteins were responsible for the majority of the connections (from 41 to 14 edges each), they were therefore considered “hub” proteins. In particular, TP53, BRCA1, and CTNNB1 were able to connect to 41, 35, and 28 melanoma proteins, respectively, thus making these proteins the most interconnected within the core-level-zero PPI network.

**Fig. 5.**
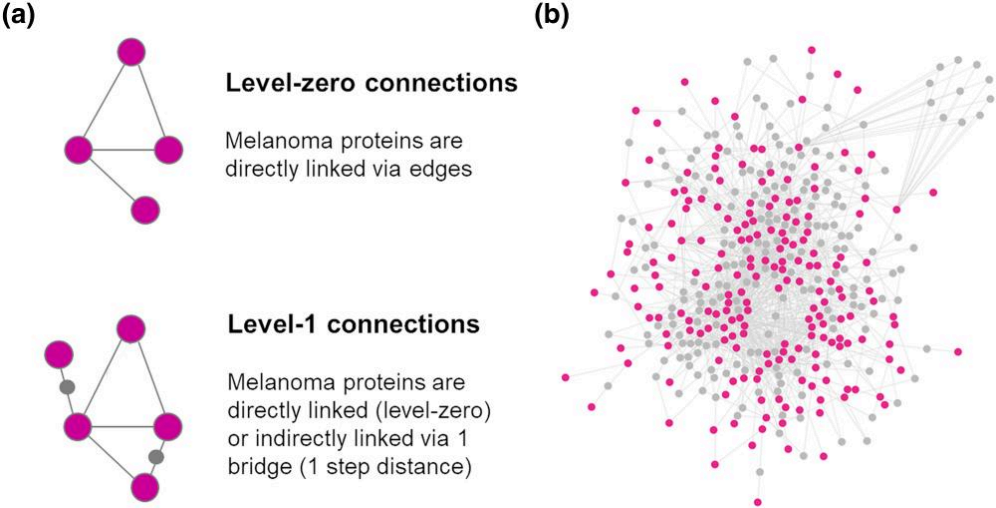
PPI network (level 1 interactions). a) Schematics of node connections within the level-zero and level-1 protein networks. b) Level-1 protein network; larger nodes in pink are the protein products of the melanoma risk genes; the nodes in gray are the bridges that are not melanoma risk proteins but mediate the connection (1 step distance) between at least 2 melanoma risk proteins in pink that do not otherwise connect directly. Picture drawn with Cytoscape, organic layout.

The first level interactions (available from peer-reviewed literature) of the 232 melanoma risk proteins (i.e. seeds) were downloaded from HIPPIE. Only interactions with a confidence score ≥ 0.72 (high confidence interactions, which corresponds to the 3rd quartile of the HIPPIE score distribution) were downloaded. The PPIs were filtered to keep only “bridge” interactors defined as interactors able to: (1) connect at least 2 of the seeds; and (2) where the 2 seeds were not already directly connected between each other (Fig. 5a). Out of the 232 melanoma risk genes, 194 were connected in a unique graph: the “core-first-level PPI network”, containing 402 unique nodes, of which 194 were seeds (melanoma risk proteins) and 208 were bridges (defined as nodes able to connect at least 2 of the seeds that were not already directly connected between each other) (Fig. 5b, Supplementary Table 7).

In summary, while the “core-level-zero network” contains the protein products of the melanoma risk genes that are connected directly to each other, the “core-first-level network” expands on the level-zero to include the protein products of the melanoma risk genes whose interaction is mediated via 1 communal interactor (i.e. bridge) that is not a melanoma risk protein itself (Fig. 5a) thus reducing the seed-centrality bias of the protein network and increasing the completeness of the melanoma interactome. Therefore, the core-first-level network will be hereafter referred to as “melanoma model”.

### Clustering analysis of the core-first-level melanoma network

**Fig. 6.**
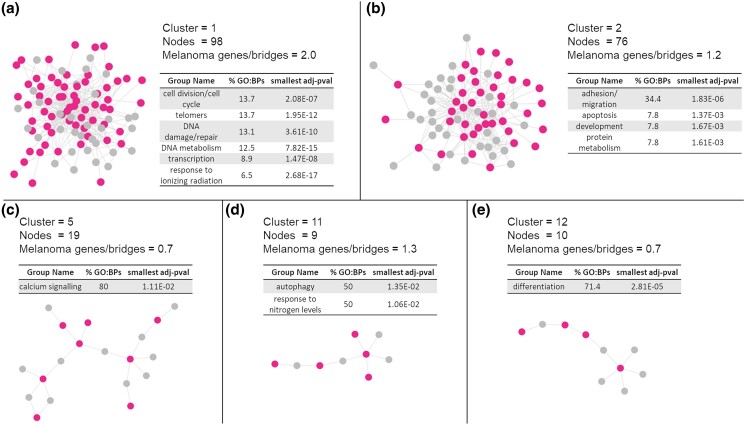
Clusters of the core-first-level melanoma network. The network clusters are reported to visualize dimension and connectivity; pink nodes are the protein products of the melanoma risk genes while the nodes in gray are the bridges that are not melanoma risk proteins but mediate the connections within the network; “melanoma genes/bridges” identifies the ratio between the 2 types of nodes. In tables, GO:BPs functional enrichment is reported (only TOP results in terms of group contribution; covering >50% of the enriched results). Significant terms were filtered and only terms with term size ≤ 300 were kept; they were further grouped and “%GO:BPs” identifies the percentage of GO:BPs in each group in comparison with the entire set of GO:BPs kept after filtering. “Smallest adj-pvalue” identifies the smallest *P*-value associated with the terms grouped. a) Cluster 1; b) cluster 2; c) cluster 5; d) cluster 11; e) cluster 12.

The core-first-level melanoma network was topologically processed (fast greedy clustering algorithm) to identify the portions of the network that can be identified as local communities within the network (i.e. clusters) based on the distance between each pair of nodes in the network. Sixteen clusters were identified (Supplementary Table 8); however, after QC based on total number of nodes in the cluster and ratio between melanoma genes and bridges in the cluster (melanoma-genes:bridges ≥ 0.7), only 5 clusters remained to be analyzed. Clusters are local communities more connected within each other in comparison with the average network connectivity (Labatut and Balasque 2012); therefore, we can assume they are likely to identify functional units within the network. We therefore conducted GO:BPs functional enrichment on the selected 5 clusters (Fig. 6). After functional enrichment processing (only GO:BPs with term size < 300 were kept for further analyses thus allowing for filtering out general terms), GO:BPs were grouped based on semantics. These groups were evaluated to calculate their contribution (in terms of total number of GO:BPs in each group) toward enrichment; the largest groups, cumulatively able to cover >50% of the enrichment, were selected as top functions descriptive of each cluster. Cluster 1 was mainly involved in processes of “DNA metabolism” (comprising “telomers”, “DNA damage/repair”, and “response to ionizing radiation”) and “cell cycle/division”; cluster 2 was involved in multiple, diverse functions with the predominant related to “cell adhesion/migration” and this might be relevant considering the migration and invasion properties of melanoma; cluster 5 was involved in “calcium signalling”; cluster 11 was involved in “response to nitrogen” and “autophagy” (of note these terms are generally connected as starvation is able to initiate and modulate autophagy); finally, cluster 12 was mainly involved in “cell differentiation” (refer to Supplementary Table 9 for the full enrichment).

### Validation of the melanoma model

**Fig. 7.**
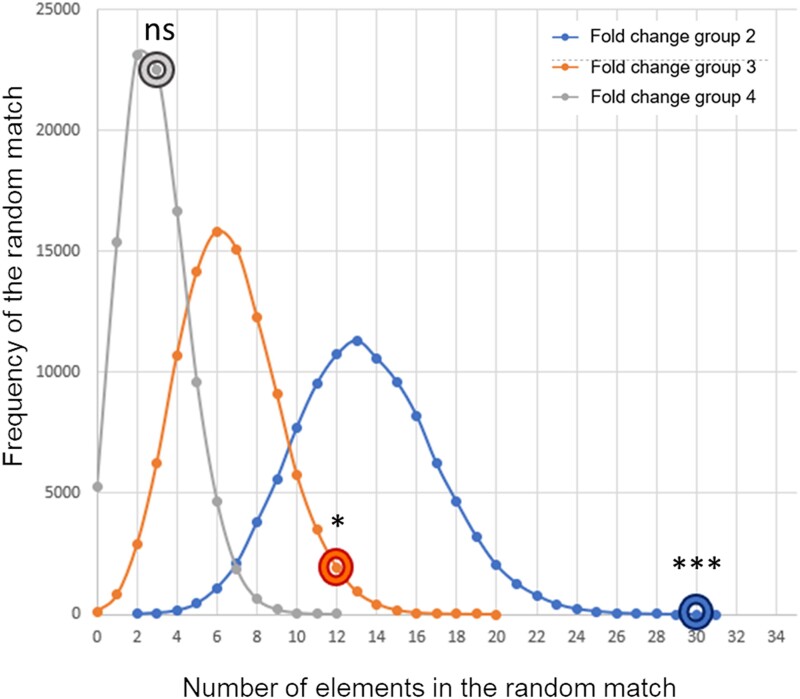
Random simulations. Distribution of the results obtained after 100,000 random experiments to simulate the overlaps between the differentially expressed genes in the fold change groups 2, 3, and 4 with the list of genes in the melanoma model. The real number of matches is reported on the random curve with the donut symbol. *P < 0.05; ***P < 0.001; ns, nonsignificant.

The GDS1375 human melanoma expression profile dataset (array data) was downloaded from GEO (Talantov et al. 2005). The signal intensity for 7 normal-skin and 45 primary malignant melanoma samples were used to calculate log2 fold change and *P*-value (Welch *t*-test) for each of the probes correctly converted to a HGNC gene symbol. All the genes with a significant fold change (Bonferroni's corrected Welch *t*-test, *P* < 2.24 × 10^−6^) in the human melanoma specimens vs normal-skin were divided into classes: fold change > 2 or <−2 (FC_group2); fold change > 3 or <−3 (FC_group3); and fold change > 4 or <−4 (FC_group4) before being overlayed over the list of genes in the melanoma model. The enrichment *P*-value was calculated by running 100,000 random simulations in which a randomly generated list of genes (with the same number of elements present in each of the fold change classes) was overlayed over the real list of genes in the melanoma model (*P*-value calculated assuming normal distribution of the random event, lower tail = false). Both the FC_group2 and FC_group3 showed a significant enrichment of genes in the melanoma model (30/402 differentially expressed genes over the total number of genes in the melanoma model, pnorm-value = 1.62 × 10^−6^; and 12/402, pnorm-value = 0.017, respectively) showing that a nonrandom portion of the genes in the melanoma model is indeed altered in expression in human malignant melanoma samples (Fig. 7 and Supplementary Fig. 1).

### DEA in harmine and berberine chloride treated human melanoma cells

We then sought to evaluate whether it was possible to use the melanoma model as a prediction tool to determine whether a phytochemical compound might be effective in interfering with the melanoma pathways. We selected 2 different compounds, harmine and berberine chloride, due to the body of literature reporting their anti-melanoma activity. They were both shown to have promising anticancer activity in murine and human melanoma cellular models (Hamsa and Kuttan 2011b, Hamsa and Kuttan 2012, Liu et al. 2018). Harmine and berberine chloride activities were tested in the SK-MEL-28 human melanoma cell line to determine the effective dose (ED) of the available batches of compounds. Cells were plated and allowed to adhere to the substrate for an overnight before being treated with harmine or berberine chloride. Cell density was assessed 72 hours from treatment using the sulforhodamine B (SRB) assay. Both harmine and berberine chloride showed cytotoxic activity (induction of cell death and/or inhibition of cell growth) as the cell density was significantly reduced upon treatment in comparison with vehicle treated controls (Supplementary Fig. 2).

In order to evaluate early gene regulatory changes induced by the treatment, SK-MEL-28 cells were exposed to the ED of both harmine and berberine chloride for 24 hours before RNA collection and sequencing. DEA was performed (SARTools DESeq2 R package) to evaluate genes that were significantly up- or down-regulated upon phytochemical treatment in comparison with vehicle treated, control cells (Supplementary Tables 10 and 11). Hierarchical clustering of the DE genes showed good clustering of the 3 conditions: controls, harmine, and berberine chloride treated cells, thus confirming minimal technical variation across experimental replicates (Supplementary Fig. 3).

**Fig. 8.**
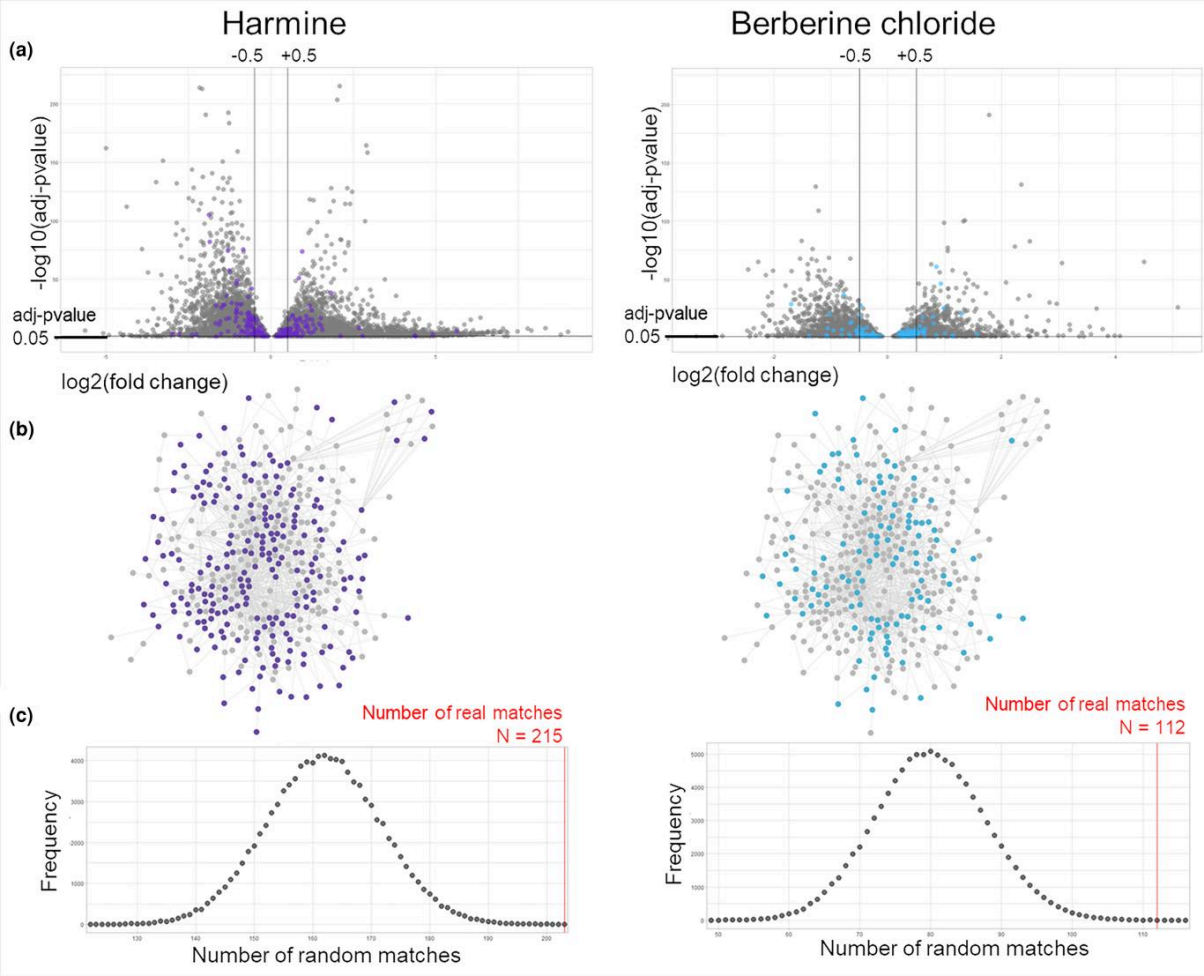
The effect of anti-melanoma compounds on the melanoma network model. a) Volcano plot representing all the genes that present with significant differential expression after harmine or berberine treatment. The differentially expressed genes that are members of the melanoma network model (i.e. core level 1 protein network) are color-coded (harmine in purple and berberine in light blue). Lines indicate adjusted *P*-value of 0.05 and log2(fold change) ± 0.5. b) Level-1 protein network; the gray nodes are not significantly altered in expression upon drug treatment, expression of nodes in purple is significantly altered after treatment with harmine, expression of nodes in light blue is significantly altered after treatment with berberine. c) Distribution of results obtained after 100,000 random simulations in which random lists of proteins simulating the melanoma model have been matched over the genes that are significantly altered in expression after harmine or berberine treatment, the number of real matches obtained with the real melanoma model are reported in red.

The genes that showed significant alteration in expression levels upon drug treatment (adjusted *P* < 0.05) were overlayed on the melanoma model and the number of overlaps was calculated. Harmine treatment induced significant modification in the expression of 53.5% of the genes involved in the melanoma model (215 over 402 genes/proteins) (Fig. 8a, Supplementary Table 12). Similarly, berberine chloride was able to alter the expression of 27.9% of the genes involved in the melanoma model (112 over 402 genes/proteins) (Fig. 8a, Supplementary Table 13). Both results were confirmed as significant (*P* = 2.3 × 10^−8^ and 2.6 × 10^−5^, respectively) upon 100,000 random simulation experiments (and assuming a normal distribution of the random events) (Fig. 8b).

**Fig. 9.**
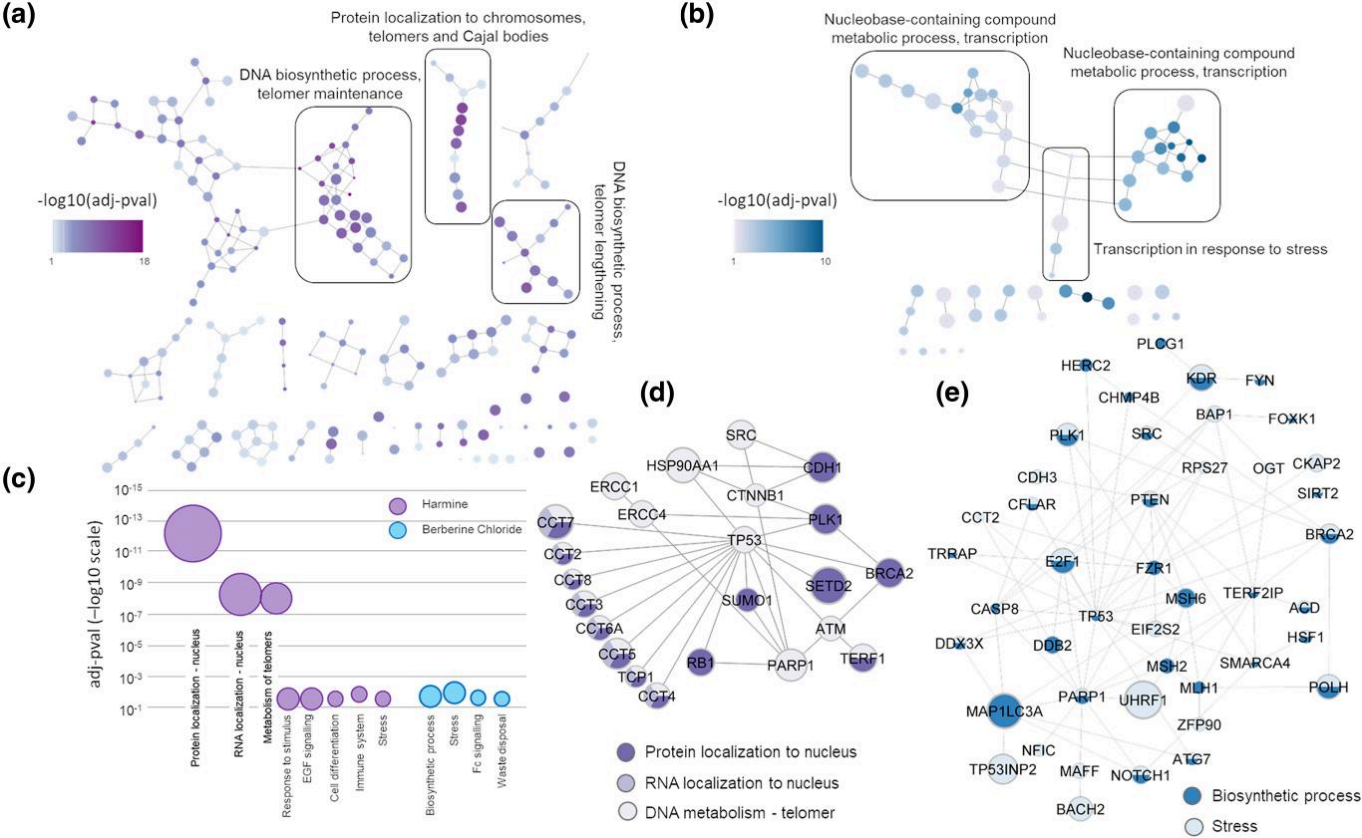
The effect of anti-melanoma compounds on the melanoma network model. Hierarchical connections of GO:BP terms after functional enrichment for the parts of the melanoma model network differentially expressed after harmine a) or berberine chloride b) treatment. Each node is a GO:BP term from enrichment [term size was kept <2,500 a) or <3,000 b) thus allowing to plot the majority of the enriched terms]. The color of the nodes represents the *P*-value, the size of the node is inversely proportional to the term size. The edges are drawn by the R package GO.db and represent parent–child terms relationships. The biggest clusters containing the larger (smallest term size) and darker (smallest *P*-value) nodes were selected. c) The functional enrichment was further processed by removing GO:BPs with term size > 50 to allow specific terms only. The GO:BPs were semantically classified into larger groups. The bubble graph represent the larger groups, the size of the circle represents the number of GO:BPs in each semantic group, plotted against the smallest *P*-value (*y*-axis in −log10 scale) within the group. Differentially expressed genes involved in the main functions identified via enrichment were extracted from the melanoma model for d) harmine and e) berberine. The largest connected clusters are shown, dimension of the node is proportional to the log2 fold change.

The 215 and 112 genes in the melanoma model showing differential expression upon harmine and berberine treatments were functionally annotated (Supplementary Tables 14 and 15) via GO:BPs enrichment and Reactome pathways enrichment (Supplementary Tables 16 and 17). The genes altered by harmine treatment showed primary involvement in GO:BPs related to “DNA metabolism of telomeres”, “RNA and protein localization to Cajal bodies”, and “to telomeres”. The genes altered by berberine hydrochloride treatment showed primary involvement in functions related to “cellular response to stress”, “regulation of transcription in response to stress”, and “biosynthesis of melanin pigments and metabolites” (Fig. 9a–c). The proteins altered in expression by drug treatment and contributing to the enrichment of these top functions were extracted and visualized (Fig. 9d and e). Reactome pathway enrichment showed a less focused type of enrichment and results were more general. Harmine related genes were involved in multiple pathway terms, mainly suggesting “immune signalling”, “DNA repair processes”, “protein folding”, “apoptosis”, and “transcription”. In the case of berberine chloride modified genes, a very limited number of pathways were enriched pointing toward “signal transduction”, “DNA repair”, “apoptosis”, and “de-ubiquitination” (Supplementary Fig. 4).

## Discussion

Complex disorders such as cancer, dementias, and cardiovascular disease are triggered by a complex set of molecular events (or risk elements) responsible for the pathogenic alteration of the cellular homeostasis. In general, none of the single events is, *per se*, strong enough to initiate and sustain the progression of the disease; however, the combination of all risk elements increases the personal chance of developing a certain disease. This peculiar nature of complex disorders makes them the perfect subject for network modeling as their molecular mechanism is better studied via a systems biology approach where multiple players are considered for their group effect rather than via a [single-cause: single-consequence] type of modeling (Vidal et al. 2011, Tomkins and Manzoni 2021).

Here we used a modeling approach where a network was built from a selection of “seeds” defined as genes relevant for melanoma. We selected genes known for being mutated in familial melanoma, genes whose somatic mutation contributes to melanoma and we prioritized risk genes from GWAS. It is important to highlight that the prioritization of candidate genes responsible for GWAS risk signals is not straightforward as GWAS pin-points loci of risk rather than causal genes. Therefore, we evaluated the recent melanoma GWAS (Landi et al. 2020) and prioritized the genes that are in physical proximity or in a LD block around the tag SNPs. Additionally, we considered those genes presenting in eQTL with the tag SNPs. Using this approach, we compiled a list of candidate genes that might be responsible for the GWAS signals. A future refinement of this prioritization would require to consider splicing QTLs and alteration in expression and co-expression changes of prioritized genes in a disease cohort. The list of GWAS candidate genes we prioritized is lengthy, as it probably contains true and false positives. In fact, while the familial and sporadic melanoma genes are almost certainly associated with melanoma, for the candidate genes prioritized using the GWAS signals, there is a degree of uncertainty as to whether they are the true molecular cause responsible for the GWAS associations (they can only be indicated as candidate genes). Only further functional studies will be able to provide a definitive answer and this uncertainty is embedded in the nature of the analytical approach used for these types of studies. The combination of familial, sporadic, and GWAS genes provided us with a comprehensive list of 232 unique genes that are genetically connected (albeit at different extents) to the risk of melanoma and represented the seed list for starting the network modeling. It was important at this stage to define the functions and pathways associated with the list of seeds and evaluate whether they were consistent with cancer and melanoma. Indeed, the most relevant and specific GO:BPs pointed toward functions related to: antigen presentation, metabolism of telomeres, DNA damage and repair, metabolism of pigments, and ageing. Similarly, the most relevant Reactome pathways pointed toward functions related to DNA repair, signaling via RAF, BRAF, RAF, and ERK, transcription, telomere associated pathways, and melanin biosynthesis. This confirmed the relevance of the seed list in the context of this study. The majority of the GO:BPs and pathways associated with the melanoma-gene list is, in fact, related to processes known for their alteration during cancer, for instance DNA damage and repair processes, the telomere maintenance that can be linked to cell ageing and their shortening due to the uncontrolled cellular replications, as well as the signaling pathways identified. Some of the terms are specifically valid in the context of melanoma, such as those referring to the metabolism of pigments in general and melanin specifically. Interestingly, antigen presentation was one of the most enriched processes following GO enrichment. New antigens are known to be presented on the cancer cell surface and this is key for the immune system to recognize and destroy cancer cells. An imbalance in this process because of disfunctions of the immune system in its ability to recognize cancerous cells, as well as the ability of tumors to escape recognition by the immune system (e.g. via antigen presentation regulation) have been linked to cancer onset and progression (Jhunjhunwala et al. 2021, Yang et al. 2023).

To build the melanoma model, we initially evaluated the protein interactions directly connecting, one to another, the protein products of the seed list. This first step assumed that if the seed genes are all associated to the same disease their protein products should be involved in a set of similar functions. Proteins that work together are likely to interact (Koh et al. 2012); and, in fact, the majority (∼67%) of the melanoma seeds were able to form direct connections between each other originating a unique, interconnected cluster whose interconnectivity was sustained by hub nodes such as TP53, BRCA1, and CTNNB1. Due to the nature of PPI studies, it is important to acknowledge that there is always an ascertainment bias associated with interactome models. Hub nodes might, for example, not be the only important, highly connected nodes within the interactome, there might be hidden hub nodes that we miss during analysis because they represent less studied genes/proteins, nodes for which information related to connectivity is not available yet in literature. On the other hand, some PPI might be false positives, due to the increased use of high-throughput techniques in the discovery of protein interactions. One way to reduce the impact of these biases might be that of “scaling-up” the model, adding levels of indirect PPIs to increase the density and completeness of the interactome/network model. We therefore expanded the level 0 network by collecting (from peer-reviewed literature) all the reported PPIs for the melanoma seeds. This step was fundamental to understand how the protein products of the melanoma risk genes are connected to other proteins within the cellular milieu, even if these additional proteins are not, per se, genetically linked to melanoma. This led to the building of the core level 1 network, containing the seeds (i.e. the protein products of the melanoma risk genes, *n* = 194, ∼84% of the seed list), their direct connections as well as the connections mediated via shared protein bridges. The core level 1 network can be therefore regarded as the overall melanoma model, the system providing a potential, in silico overview over the core machinery involved in the melanoma processes. As such, it holds the potential to be used for predictions on functions involved in melanoma and as well as potential intervention sites. We topologically analyzed this core melanoma network to verify if density clusters could be identified. Topological analysis on PPIs network is intrinsically bias as PPI networks are by definition incomplete because, as mentioned before, not all the protein interactions has been discovered yet. This leads to an under-estimation of the connectivity within the network. However, and considering this caveat, we identified 16 clusters, 5 of which survived QC. Functional annotation of these clusters using GO:BPs suggested pertinent functions in the context of cancer, melanoma, and metastatic processes thus confirming the relevance of the core melanoma network as in silico model of the melanoma interactome.

To verify the relevance to disease for the in silico melanoma model we generated, we made use of a GEO dataset (Talantov et al. 2005) reporting the expression profiles of different human samples collected from normal-skin and malignant melanoma. We were able to show that a significant part of the in silico melanoma model was indeed altered in expression (2- or 3-fold change) when the expression profiles of melanoma vs control human samples were compared. This suggests that the in silico melanoma model we generated might hold relevance in the disease scenario and it could be therefore used as a modeling tool to dissect the mechanisms and gene regulatory events in the context of melanoma. It is important to remember that this all sits in the realm of in silico biology and therefore, any conclusion has still to be functionally validated.

To exemplify a potential application of the melanoma network model built so far, we assessed whether it could be used to predict the activity of anti-melanoma compounds. The hypothesis was that a compound able to modify melanoma cell growth or interfere with melanoma progression should show an impact on a significant portion of the proteins/genes involved in the melanoma model. The impact can be at different levels, we decided to focus on the expression level of the melanoma model. We treated SK-MEL-28 human melanoma cells with a toxic dose of harmine and berberine chloride before collecting the RNA and evaluate differential gene expression. Both compounds were able to significantly alter the expression of a large set of genes in comparison with untreated controls. Interestingly, a significant number of differentially expressed genes were, as hypothesized, part of the melanoma model suggesting that this might explain the anti-melanoma properties of these metabolites. The melanoma model, in fact, was built as a protein network to recapitulate (at least in part) the molecular pathways driven by the melanoma genes considered as a collective unit. The hypothesis is that, under physiological condition, these pathways are balanced, while pathogenic mutations can perturb the correct relationship across the different network elements, thus unbalancing the model and causing disease. The phytochemical treatment was able to directly or indirectly affect the expression levels of a significant portion of elements in the melanoma model and, by so doing, phytochemicals were actively perturbing the flux of information within the melanoma network. This might justify their anticarcinogenic activity detected in vitro. The elements altered by harmine were mainly involved in functions related with DNA metabolism, maintenance of telomeres and relocation of proteins and RNA to the nucleus, telomeres, and Cajal bodies. These functions were sustained by an altered network centered on TP53 and PARP1 (connection hubs) with the largest fold change experienced by SETD2, HSP90AA1, and CCT7. However, berberine seemed to affect less specific pathways, mostly related with cellular responses to stress, with an altered network centered on TP53, PARP1, FZR1, PTEN, E2F1, and TERF2IP; with the largest fold change experienced by UHRF1 and MAP1LC3A. It is important to note that genes/proteins are generally involved in multiple functions, therefore it is not unusual to retrieve some “back-ground” noise while running functional enrichment. On a similar note, due to the way the ontologies of reference are built, it is not always possible to retrieve specific/root terms while general functions (less specific) are generally abundant in enrichment analyses. The information we obtained might be useful to guide further functional research aimed at characterizing harmine and berberine modes of action, but it also identifies sensitive elements within the network, these being the molecular connections altered by harmine and berberine, compounds with a proven anti-melanoma activity. These elements can be suggested as intervention points to prioritize other molecules known for their activity toward these molecular targets but whose anti-melanoma properties have not yet been evaluated.

Systems biology approaches can, therefore, constitute a guidance for functional research as a way to triage interventions for complex systems/disorders. In this manuscript, we have used a limited model, centered on prioritized genes for melanoma and developed as PPI network. Future models would benefit from the integration of multiple data such as gene–gene interactions and regulatory connections in order to embed within the model additional layers of complexity which are fundamental for describing the real biological system.

In conclusion, we here presented a computational pipeline leading to a network model of the gene/protein connections in cutaneous melanoma. The use of this model network could aid in the identification of the global molecular pathways relevant for disease and thus in prioritizing specific points of intervention, and in the screening of potentially active compounds. The analysis of the specific melanoma model alterations can, in fact, help in understanding the molecular role of active compounds, thus guiding further verification via functional research. Finally, with this work, we further substantiated the anti-melanoma activity of harmine and berberine chloride, highlighting the need for more targeted functional studies to advance the use of these phytochemicals to the clinical practice.

## Supplementary Material

jkad274_Supplementary_Data

## Data Availability

Data and scripts are available within the supplementary tables and files. RNAseq data generated within this project are available in GEO, GSE246383. Dataset: Dmitri Talantov, Abhijit Mazumder, Jack X Yu, Thomas Briggs, Yuqiu Jiang, John Backus, David Atkins, Yixin Wang, 2005, Cutaneous malignant melanoma, GEO, GDS1375. Supplemental material available at G3 online.
