# Supplementary material for: Active natural compounds perturb the melanoma risk-gene network
Source: G3 (Bethesda). 2023 Nov 30;14(2):jkad274. doi: 10.1093/g3journal/jkad274 (PMC10849364; doi:10.1093/g3journal/jkad274)
Supplement: jkad274_Supplementary_Data [file jkad274_supplementary_data.zip › Supplemental_Figures_G3-2023-404601.pptx]

## Slide 1
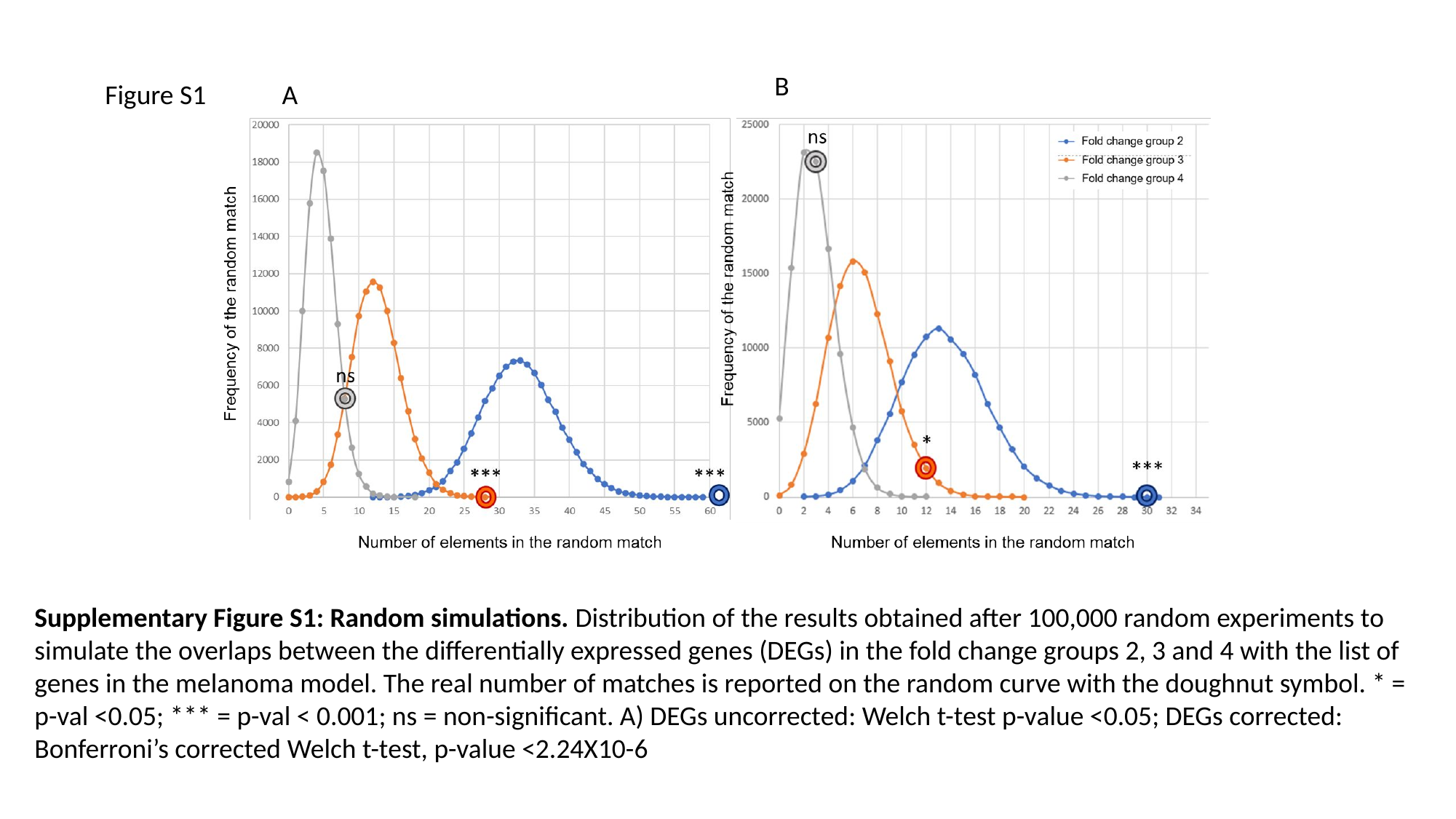

B
Figure S1
A
Supplementary Figure S1: Random simulations. Distribution of the results obtained after 100,000 random experiments to simulate the overlaps between the differentially expressed genes (DEGs) in the fold change groups 2, 3 and 4 with the list of genes in the melanoma model. The real number of matches is reported on the random curve with the doughnut symbol. * = p-val <0.05; *** = p-val < 0.001; ns = non-significant. A) DEGs uncorrected: Welch t-test p-value <0.05; DEGs corrected: Bonferroni’s corrected Welch t-test, p-value <2.24X10-6

## Slide 2
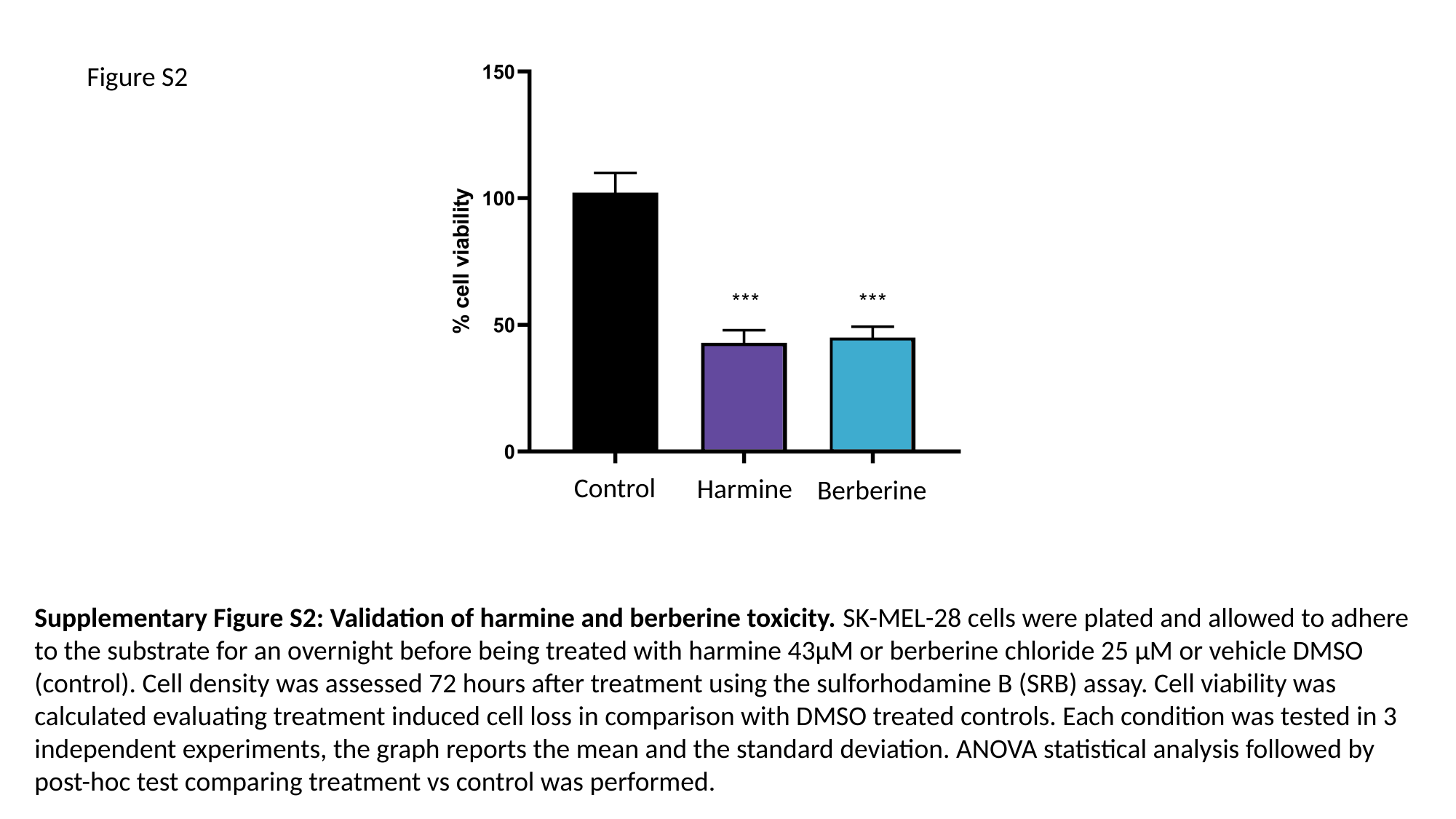

Figure S2
Control
Harmine
Berberine
Supplementary Figure S2: Validation of harmine and berberine toxicity. SK-MEL-28 cells were plated and allowed to adhere to the substrate for an overnight before being treated with harmine 43μM or berberine chloride 25 μM or vehicle DMSO (control). Cell density was assessed 72 hours after treatment using the sulforhodamine B (SRB) assay. Cell viability was calculated evaluating treatment induced cell loss in comparison with DMSO treated controls. Each condition was tested in 3 independent experiments, the graph reports the mean and the standard deviation. ANOVA statistical analysis followed by post-hoc test comparing treatment vs control was performed.

## Slide 3
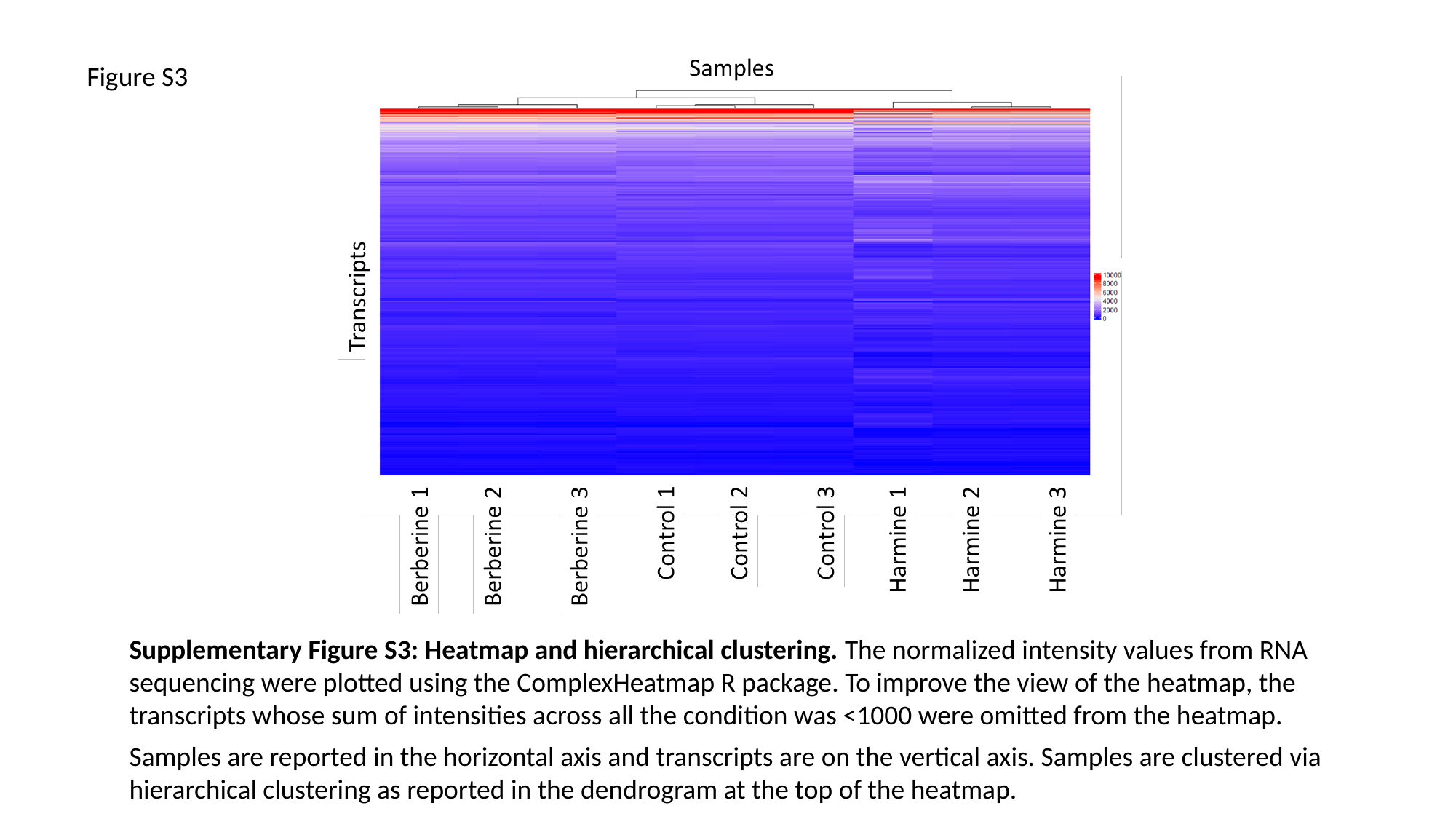

Figure S3
Supplementary Figure S3: Heatmap and hierarchical clustering. The normalized intensity values from RNA sequencing were plotted using the ComplexHeatmap R package. To improve the view of the heatmap, the transcripts whose sum of intensities across all the condition was <1000 were omitted from the heatmap.
Samples are reported in the horizontal axis and transcripts are on the vertical axis. Samples are clustered via hierarchical clustering as reported in the dendrogram at the top of the heatmap.
S2. The normalized intensity values were plotted with the ComplexHeatmap R package. To improve the view of the heatmap, the transcripts whose sum of intensities across all the condition was <1000 were omitted from the heatmap

## Slide 4
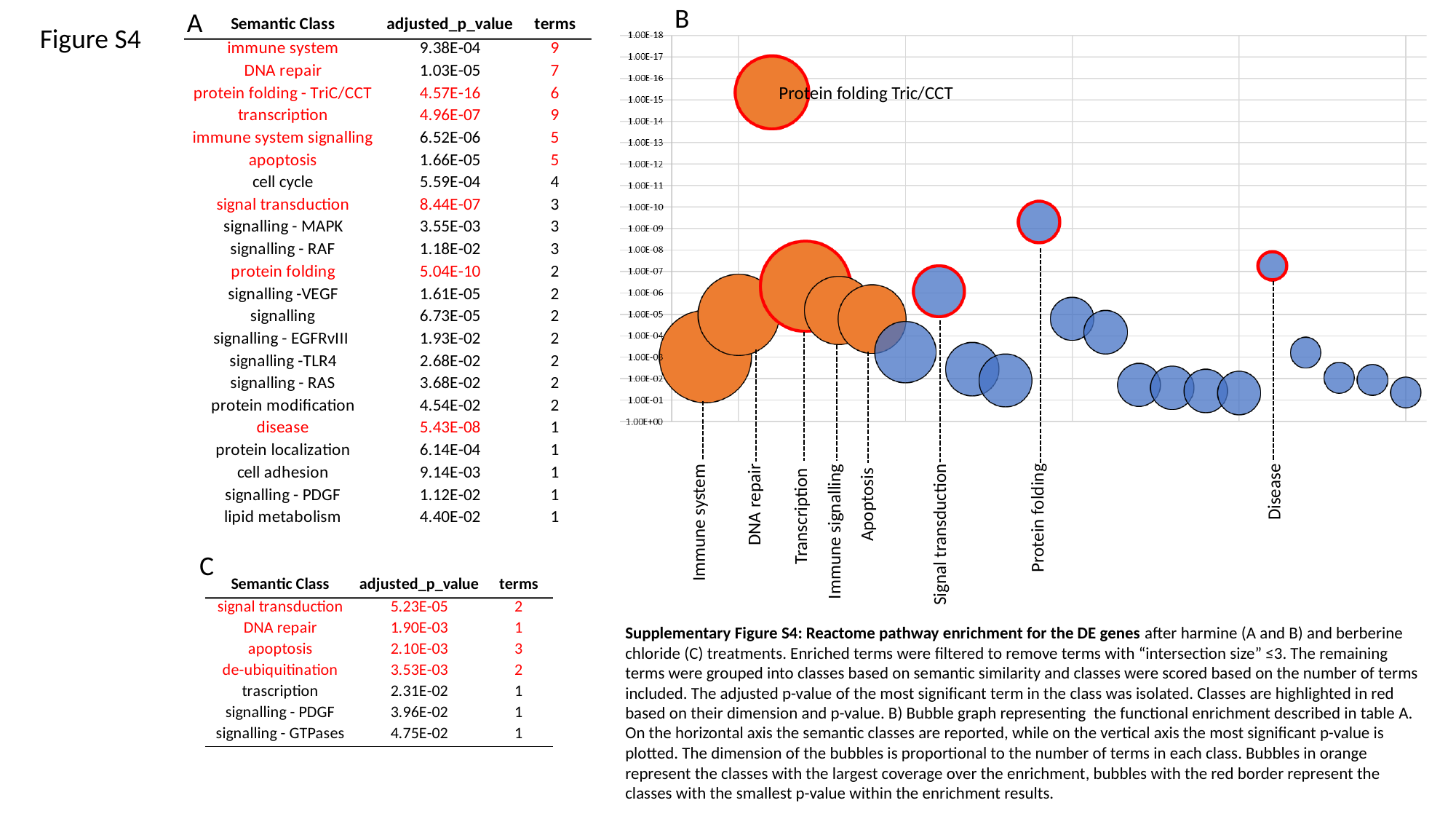

A
B
Figure S4
Protein folding Tric/CCT
Disease
Apoptosis
DNA repair
Transcription
Protein folding
Immune system
Immune signalling
Signal transduction
C
Supplementary Figure S4: Reactome pathway enrichment for the DE genes after harmine (A and B) and berberine chloride (C) treatments. Enriched terms were filtered to remove terms with “intersection size” ≤3. The remaining terms were grouped into classes based on semantic similarity and classes were scored based on the number of terms included. The adjusted p-value of the most significant term in the class was isolated. Classes are highlighted in red based on their dimension and p-value. B) Bubble graph representing the functional enrichment described in table A. On the horizontal axis the semantic classes are reported, while on the vertical axis the most significant p-value is plotted. The dimension of the bubbles is proportional to the number of terms in each class. Bubbles in orange represent the classes with the largest coverage over the enrichment, bubbles with the red border represent the classes with the smallest p-value within the enrichment results.
